# Confirmed *Bacillus anthracis* Infection among Persons Who Inject Drugs, Scotland, 2009–2010

**DOI:** 10.3201/eid2009.131481

**Published:** 2014-09

**Authors:** Malcolm Booth, Lindsay Donaldson, Xizhong Cui, Junfeng Sun, Stephen Cole, Susan Dailsey, Andrew Hart, Neil Johns, Paul McConnell, Tina McLennan, Benjamin Parcell, Henry Robb, Benjamin Shippey, Malcolm Sim, Charles Wallis, Peter Q. Eichacker

**Affiliations:** Glasgow Royal Infirmary, Glasgow, Scotland, UK (M. Booth, L. Donaldson, A. Hart);; National Institutes of Health, Bethesda, Maryland, USA (X. Cui, J. Sun, P.Q. Eichacker);; Ninewells Hospital, Dundee, Scotland, UK (S. Cole, B. Parcell);; Victoria Infirmary, Glasgow (S. Dailsey);; Queen Margaret and Victoria Hospitals, Dumfermline, Scotland, UK (N. Johns, B. Shippey);; Crosshouse Hospital, Kilmarnock, Scotland, UK (P. McConnell);; Hairmyres Hospital, East Kilbride, Scotland, UK (T. McLennan);; Forth Valley Royal Hospital, Larbert, Scotland, UK (H. Robb);; Western Infirmary, Glasgow (M. Sim);; Western General Hospital, Edinburgh, Scotland, UK (C. Wallis)

**Keywords:** anthrax, persons who inject drugs, PWID, outbreak, bacteria, Scotland, United Kingdom, Bacillus anthracis

## Abstract

Patients who died had an increased sequential organ failure assessment score and need for vasopressors.

## Medscape CME Activity

Medscape, LLC is pleased to provide online continuing medical education (CME) for this journal article, allowing clinicians the opportunity to earn CME credit. 

This activity has been planned and implemented in accordance with the Essential Areas and policies of the Accreditation Council for Continuing Medical Education through the joint providership of Medscape, LLC and Emerging Infectious Diseases. Medscape, LLC is accredited by the ACCME to provide continuing medical education for physicians.

Medscape, LLC designates this Journal-based CME activity for a maximum of 1.0 ***AMA PRA Category 1 Credit(s)^TM^***. Physicians should claim only the credit commensurate with the extent of their participation in the activity.

All other clinicians completing this activity will be issued a certificate of participation. To participate in this journal CME activity: (1) review the learning objectives and author disclosures; (2) study the education content; (3) take the post-test with a 75% minimum passing score and complete the evaluation at http://www.medscape.org/journal/eid; (4) view/print certificate.

**Release date: August 15, 2014; Expiration date: August 15, 2015**

## Learning Objectives

Upon completion of this activity, participants will be able to:

- Distinguish patient factors associated with a higher risk for mortality from injection anthrax
- Evaluate variables at patient presentation associated with a higher risk for death from injection anthrax
- Assess medical treatment variables associated with a higher risk for death from injection anthrax
- Analyze hospital events associated with a higher risk for mortality from injection anthrax.

## CME Editor

**Karen L. Foster, **Technical Writer/Editor, *Emerging Infectious Diseases. Disclosure: Karen L. Foster has disclosed no relevant financial relationships.*

## CME Author

**Charles P. Vega, MD, **Clinical Professor of Family Medicine, University of California, Irvine. *Disclosure: Charles P. Vega, MD, has disclosed the following financial relationships: served as an advisor or consultant for McNeil Pharmaceuticals.*

## Authors

*Disclosures:* ***Malcolm Booth, MBChB, FRCA, MPhil, FFICM; Lindsay Donaldson, MB ChB, FRCA, FFICM; Xizhong Cui, MD, PhD; Junfeng Sun, PhD; Stephen Cole, BSc(Hons), MBChB, FRCA, FFICM, FRCP; Susan Dailsey, MBChB; Andrew Hart, BSc, MBChB, MD, PhD, MRCS, AFRCS, FRCS(plast); Neil Johns, MBBS, BSc; Paul McConnell, MBChB(Hons), FRCA, EDIC, FFICM; Tina McLennan, MBChB, FRCA; Benjamin Parcell, MBChB, FRCPath; Henry Robb, MBChB, FRCA, MS, FFICM; Charles Wallis, MBChB, FRCA, FFICM;*** *and* ***Peter Q. Eichacker, MD,*** *have disclosed no relevant financial relationships.* ***Benjamin Shippey, FRCPE, FFICM, FRCA, BM, BS, BMedSci,*** *has disclosed the following relevant financial relationships: served as an advisor or consultant for and received grants for clinical research from Aircraft Medical.* ***Malcolm Sim, BSc(Hons), MBChB, FRCA, FRCP, FFICM, EDIC,*** *has disclosed the following relevant financial relationships: received grants for clinical research from Roche/Wyeth Pharmaceuticals Inc.*
