# Supplementary material for: Confirmed Bacillus anthracis Infection among Persons Who Inject Drugs, Scotland, 2009–2010
Source: Emerg Infect Dis. 2014 Sep;20(9):1452–63. doi: 10.3201/eid2009.131481 (PMC4178387; doi:10.3201/eid2009.131481)
Supplement: Related texts

# Review article

## Abstract

In Scotland, the 2009 outbreak of *Bacillus anthracis* infection among persons who inject drugs resulted in a 28% death rate. To compare nonsurvivors and survivors, we obtained data on 11 nonsurvivors and 16 survivors. Time from *B. anthracis* exposure to symptoms or hospitalization and skin and limb findings at presentation did not differ between nonsurvivors and survivors. Proportionately more nonsurvivors had histories of excessive alcohol use (p = 0.05) and required vasopressors and/or mechanical ventilation (p<0.01 for each individually). Nonsurvivors also had higher sequential organ failure assessment scores (mean + SEM) (7.3 + 0.9 vs. 1.2 + 0.4, p<0.0001). Antibacterial drug administration, surgery, and anthrax polyclonal immune globulin treatments did not differ between nonsurvivors and survivors. Of the 14 patients who required vasopressors during hospitalization, 11 died. Sequential organ failure assessment score or vasopressor requirement during hospitalization might identify patients with injectional anthrax for whom limited adjunctive therapies might be beneficial.

*Bacillus anthracis* infection in humans has typically been classified as cutaneous, gastrointestinal, or inhalational on the basis of the bacterium’s route of entry (*1*). However, in Scotland, United Kingdom, during 2009–2010, a total of 47 patients had confirmed *B. anthracis* soft tissue infection related to injection of contaminated heroin (*2*,*3*). This form of *B. anthracis* infection appears to be distinct from cutaneous disease and has been termed “injectional” anthrax (*2*–*5*). In addition to confirmed cases, 35 probable and 37 possible cases in Scotland, 5 confirmed cases in England, and 2 confirmed cases in Germany also were identified. This initial outbreak ended in late 2010, but since the summer of 2012, new cases have been reported in the United Kingdom and Europe (*3*,*4*). Although 1 case of injectional anthrax was recognized in Norway in 2001, the patients in 2009–2010 constitute the first large outbreak of this newly recognized and poorly characterized form of anthrax (*5*).

Health Protection Scotland (HPS) has published epidemiologic analyses of the 2009–2010 outbreak (*3*,*6*,*7*). Among other findings, analysis suggested associations between longer injecting histories, opioid substitution therapy, and alcohol use and risk for *B. anthracis* infection in persons who inject drugs (PWID) (*6*). Several case reports from the outbreak also have been published (*8*–*12*), but they did not include systematic examinations of the physical, laboratory, and surgical findings or of therapies administered. Notably, although 13 of the 47 persons from Scotland who had confirmed cases died, no published report has compared findings in survivors and nonsurvivors. Such a comparison is needed for the prognosis and management of future cases. We therefore sent a questionnaire regarding these issues to clinicians who had treated PWIDs in whom *B. anthracis* infection was confirmed in Scotland during the outbreak.

## Methods

### Approval

We used data collected during routine hospital care of patients. Patient identifiers were removed from data before analysis. Because of the retrospective nature of the study and the anonymity of data, the West of Scotland Research Ethics Service (Glasgow, Scotland, UK) and the Office of Human Subjects Research from the Clinical Center at the National Institutes of Health (Bethesda, MD, USA) exempted the study from formal review.

### Data Collection

We developed an electronic questionnaire that requested information in several areas. These were: general information (i.e., age, sex, and medical and drug histories); current illness; data at the time the patient sought care, including skin and limb findings, vital signs, laboratory findings, and diagnosis (i.e., was anthrax infection or sepsis initially suspected?); medical and surgical treatments at the time the patient sought care or later (including antibacterial drugs; need for hemodynamic, respiratory, renal replacement, or blood product support; and use of anthrax immune globulin, a polyclonal antibody produced by Cangene [Winnipeg, MB, Canada] and made available by the Centers for Diseases Control and Prevention [Atlanta, GA, USA]; microbiological data supporting the diagnosis of anthrax infection and the time at which the diagnosis was confirmed; surgical findings; other procedures performed during hospitalization; levels of organ injury based on the sequential organ failure assessment (SOFA) score; and outcomes, including survival, time in the intensive care unit (ICU), and total time in hospital.

During this anthrax outbreak, HPS identified 13 hospitals to which the 47 patients with confirmed anthrax were admitted (*3*). In March 2012, two of the authors (L.D. and M.G.B.), who were members of the HPS anthrax outbreak control team, asked physicians known to have treated persons with confirmed anthrax to complete the questionnaire. The questionnaire was sent to these physicians in early April 2012. In June 2012, physicians who had not yet returned it were asked to do so. Data from all questionnaires completed by the end of August 2012 were analyzed. We received no additional questionnaires after August 2012. Contributors were subsequently contacted to clarify missing or unclear responses.

### Data Analysis

A variable was reported on only if >50% (i.e., >14) of questionnaires provided definitive data. SOFA score was calculated as previously described for each patient for whom data were obtained within the initial 24 hours after they sought care (*13*). Measures of time were expressed as medians (interquartile range [IQR]). We used Wilcoxon rank sum test to compare these times between survivors and nonsurvivors. Categorical data (i.e., chief complaints, types of initial surgery and gross tissue findings, skin and limb findings, and treatments) were analyzed with Fisher exact or χ^2^ test, where applicable, and continuous data (i.e., laboratory data, age, vital signs, and SOFA score) were examined with 1-way ANOVA (analysis of variance).

## Results

### Initial Findings

We received data on 27 confirmed cases from the outbreak: 16 of the 33 survivors and 11 of the 14 nonsurvivors from 10 of 13 hospitals that admitted PWID with outbreak-associated anthrax. The median (IQR) times (days) from exposure to onset of symptoms and from onset of symptoms to hospital admission were 1 (0–4) and 2 (2–4), respectively, and these did not differ significantly between survivors (1 [0–4.5] and 3 [2–5] days) and nonsurvivors (1 (0–2] and 2 [2– 2] days) (p = 0.90 and p = 0.19, respectively) (Figure 1).

**Figure 1:** Key events for 16 survivors (A) and 11 nonsurvivors (B) in an outbreak of *Bacillus anthracis* infection in persons who inject drugs, Scotland, UK, 2009–2010. Patients are numbered in the order in which they sought care. Time period is from patients’ suspected exposure to contaminated heroin to their discharge from hospital or to death. Day 0 is day of hospital admission. ICU, intensive care unit.

Mean (+ SEM) age of patients was 34.5 + 1.7 years. Nonsurvivors tended to be older than survivors (38.2 + 2.8 vs. 31.9 + 1.9, p = 0.07). The proportion who were male did not differ significantly (9 [56%] survivors vs. 9 [82%] nonsurvivors, p = 0.23). Although history of excessive alcohol use was higher in nonsurvivors than in survivors (4 [80%] of 5 vs. 3 [23%] of 13, p = 0.05), tobacco use and suspected injection sites (arm, groin, buttock, or leg) and routes (intravenous or intramuscular) of contaminated drug injection did not differ significantly (p>0.19 for all, data not shown). HIV infection status was available for only 10 patients (4 nonsurvivors), and hepatitis C infection status was available for only 12 patients (5 nonsurvivors). One survivor was HIV positive, and 4 survivors and 5 nonsurvivors had histories of hepatitis C virus infection. Although the questionnaire requested other medical history (e.g., hepatitis B virus infection status), these data were not provided in sufficient numbers for analysis.

Eleven (69%) survivors and 2 (18%) nonsurvivors had only localized skin or limb symptoms (i.e., pain, swelling, erythema, exudate); 5 (31%) survivors and 4 (36%) nonsurvivors had both localized and generalized symptoms (i.e., fever, confusion, seizures, abdominal pain, fatigue, malaise, sweating, headache), and 0 survivors and 5 (45%) nonsurvivors had only generalized symptom (Table 1). The proportion of nonsurvivors who had only a localized or only a generalized complaint was less (p = 0.02) and greater (p = 0.006) than the proportion of survivors.

**Table 1:** Signs and symptoms prompting hospital visits for persons who inject drugs who had *Bacillus anthracis* Infection, Scotland, UK, 2009–2010

Patient no.*	Signs and symptoms	Degree of complaint
Survivor		
1	Pain, swelling, exudate of left groin, fever, sweating	Local, generalized
2	Pain of left buttock	Local
3	Swelling, erythema, exudate of left forearm	Local
4	Abscess of left thigh, malaise, pallor, fainting	Local, generalized
5	Pain, swelling, exudate of right antecubital fossa	Local
6	Pain, erythema, swelling of right antecubital fossa	Local
7	Pain of left buttock radiating to groin, fever, chills	Local, generalized
8	Swelling of left arm	Local
9	Pain, swelling of right arm	Local
10	Pain, swelling of right arm	Local
11	Pain, swelling of left leg	Local
12	Pain, swelling of left groin, headache, photophobia, fever	Local, generalized
13	Pain, swelling of left testis and scrotum	Local
14	Swelling, erythema of right hand	Local
15	Pain, swelling of right hand to elbow	Local
16	Swelling and exudate of left groin, fever	Local, generalized
Nonsurvivor		
1	Pain, swelling, erythema of left thigh, fever, abdominal pain	Local, generalized
2	Headache followed by delirium	Generalized
3	Pain of right thigh	Local
4	Pain of right buttock, chills, malaise	Local, generalized
5	Swelling of right arm, seizure	Local, generalized
6	Pain, erythema of right groin increasing in area	Local
7	Confusion, lethargy, malaise	Generalized
8	Pain of right hip, abdominal pain, seizures	Local, generalized
9	Abdominal pain, fever	Generalized
10	Fainting	Generalized
11	Fatigue, malaise	Generalized

*Patients are numbered in the order in which they sought care during the outbreak.

At the time they sought care, nonsurvivors had lower temperatures and systolic blood pressures and higher respiratory rates than survivors (p<0.01) (Figure 2), but other vital signs (mean + SEM), including diastolic blood pressure (68 + 4 vs. 61 + 5 mm Hg), heart rate (111 + 5 vs. 118 + 8 beats per minute), and capillary refill (2.6 + 0.4 vs. 3.6 + 0.5 seconds) did not differ significantly. Four nonsurvivors, but only 1 survivor, had a temperature <36°C when they sought care, whereas 1 nonsurvivor and 7 survivors had temperatures >38°C. Also, whereas 4 nonsurvivors had systolic blood pressures <90 mm Hg, no survivor did. The Glasgow coma score recorded during the initial 24 hours was lower for nonsurvivors than for survivors (p = 0.008) (Figure 2). The proportion of survivors and nonsurvivors for whom specific skin and limb findings were available did not differ significantly (p>0.19 for all) (Table 2).

**Figure 2:** Physical examination findings for persons who inject drugs and were part of an outbreak of *Bacillus anthracis* infection, Scotland, UK, 2009–2010. Included are data from 27 patients for whom data were available. Median (interquartile range) for temperature (A), systolic arterial blood pressure (B), respiratory rate (C), and Glasgow coma scores (D) were recorded during the first 24 hours after patients sought care. Horizontal black lines indicate median values; lower and upper boundaries indicate the 25% and 75% ranges. BPM, breaths per minute.

**Table 2:** Clinical and laboratory findings, confirmatory laboratory results, and therapies administered during hospitalization of persons who inject drugs and had *Bacillus anthracis* infection, Scotland, UK, 2009–2010*

Finding or test	Overall*	Survivor	Nonsurvivor
Skin/limb findings			
Localized edema	22/24 (92)	14/15 (93)	8/9 (89)
Local pain	19/21 (90)	14/15 (93)	5/6 (83)
Local erythema	20/24 (83)	13/16 (81)	7/8 (88)
Skin lesion	19/26 (73)	11/16 (69)	8/10 (80)
Limb pain	12/22 (55)	10/16 (63)	2/6 (33)
Limb edema	13/25 (52)	10/16 (63)	3/9 (33)
Limb mottling	10/24 (42)	5/15 (33)	5/9 (56)
Exudate	5/22 (23)	4/16 (25)	1/6 (17)
Distant edema	4/21 (19)	3/13 (23)	1/8 (13)
Ulcer	4/23 (17)	3/15 (20)	1/8 (13)
Eschar	4/24 (17)	3/16 (19)	1/8 (13)
Chemistry, hematology, and arterial blood gas results (reference)†			
Potassium, mmol/L (3.5–5.3 mmol/L)	4.2 + 0.2	3.9 + 0.1	4.5 + 0.4
Chloride, mmol/L (96–108 mmol/L)	98 + 2	101 + 2	95 + 3
Creatinine, μmol/L (40–130 μmol/L)	108 + 14	93.4 + 16.1	130 + 24
Blood urea nitrogen, mmol/L (2.5–7.8 mmol/L)	8.3 + 1.6	7.3 + 2.5	9.8 + 1.7
Glucose, mmol/L (3.6–6.0 mmol/L)	8.9 + 1.0	7.3 + 1.4	10.4 + 1.2
Alkaline phosphatase, U/L (30–130 U/L)	113 + 14	106 + 15	121 + 27
Alanine aminotransaminase, U/L (<50 U/L)	44 + 15	53 + 33	37 + 6
Total protein, g/L (60–80 g/L)	61 + 3	65 + 3	55 + 5
Leukocyte × 10^9^/L (4–11 × 10^9^/L)	16.7 + 1.5	14.7 + 1.5	18.3 + 2.8
Lymphocyte, % (18%–44%)	0.15 + 0.02	0.16 + 0.03	0.13 + 0.02
Fibrinogen, g/L (2–4.10 g/L)	1.8 + 0.2	2.0 + 0.4	1.5 + 0.2
Hydrogen ion concentration, nmol/L (35–50 nmol/L)	43 + 2	40 + 3	45 + 3
PaO_2_, kPa (9–20 kPa)	21 + 3	18 + 4	23 + 4
PaCO_2_, kPa (3.5–6.5 kPa)	5.1 + 0.5	6.0 + 0.5	4.6 + 0.5
Lactate, mmol/L (<2 mmol/L)	3.8 + 1.0	1.7 + 0.4	5.2 + 1.5
Confirmatory laboratory results			
Blood culture	14/26 (54)	7/15 (47)	7/11 (64)
Wound culture	4/12 (33)	4/10 (40)	0/2 (0)
Tissue culture	10/14 (71)	5/8 (63)	5/6 (83)
PCR	12/20 (60)	5/12 (42)	7/8 (88)
Protective antigen antibody test	12/17 (71)	9/11 (82)	3/6 (50)
Lethal factor antibody test	10/18 (56)	8/12 (67)	2/6 (33)
Protective antigen ELISA	10/15 (67)	5/10 (50)	5/5 (100)
Lethal factor ELISA	8/13 (62)	4/9 (44)	4/4 (100)
Tissue Immunohistochemistry	3/3 (100)	2/2 (100)	1/1 (100)
Therapies administered			
Packed erythrocytes	13/22 (59)	7/13 (54)	6/9 (67)
Fresh frozen plasma	13/22 (59)	6/13 (46)	7/9 (78)
Platelets	10/27 (37)	4/16 (25)	6/11 (55)
Cryoprecipitate	3/20 (15)	1/12 (8)	2/8 (25)
Renal replacement therapy	7/26 (27)	3/15 (20)	4/11 (36)
Pleural drainage	4/27 (15)	3/16 (19)	1/11 (9)
Peritoneal drainage	3/25 (12)	0/15 (0)	3/10 (3)

*No. (%) patients noted to have the finding/total no. patients for whom data were provided, except for chemistry, hematology, and arterial blood gas results, for which values are indicated. †Mean ± SEM.

Nonsurvivors had lower serum sodium, corrected calcium, albumin, and platelet levels and higher bilirubin, percentage circulating neutrophils, hemoglobin concentration, international normalized ratio, prothrombin time, partial thromboplastin time, and C-reactive protein levels (p<0.05) (all shown in Figure 3 except prothrombin time, which was median [IQR] 12 [11–13] vs.15 [13.5–16.1]). For a greater proportion of nonsurvivors than survivors, arterial blood gases were measured when they sought care (10 [91%] vs. 6 [38%], p = 0.008). In patients with for whom the following values were measured, nonsurvivors had lower bicarbonates and greater base deficits (p = 0.02). Other laboratory data did not differ significantly between survivors and nonsurvivors (Table 2).

**Figure 3:** Laboratory findings for persons who inject drugs and were part of an outbreak of *Bacillus anthracis* infection, Scotland, UK, 2009–2010. Included are the 27 patients for whom data were available. Shown are median (interquartile range) for levels of serum sodium (A), corrected calcium (B), total bilirubin (C), albumin (D), base deficit (E), bicarbonate (F), percentage neutrophil (G), hemoglobin (H), and platelets (I); international normalized ratio (INR) (J); partial thromboplastin times (PTT) (K); and C-reactive protein levels (L). Horizontal black lines indicate median values; lower and upper boundaries indicate 25% and 75% ranges.

### Clinical Impression, Treatment, and SOFA Score at Presentation

Sepsis was noted in a greater proportion of nonsurvivors than survivors at presentation (7 [70%] vs. 3 [19%], p = 0.02), but *B. anthracis* infection was not (5 [46%] vs. 12 [75%], p = 0.22). All patients were initially treated with at least 1 antibacterial drug (Table 3). Neither type nor number (3.8 + 0.3 vs. 3.6 + 0.7, respectively) of antibacterial drugs received differed significantly between survivors and nonsurvivors (p>0.06). Among survivors, 75% received ciprofloxacin and 88% received clindamycin; among nonsurvivors, 55% and 64% received these drugs, respectively. More nonsurvivors than survivors received vasopressors, oxygen support, mechanical ventilation, and corticosteroids (all p<0.002) (Figure 4) but not undergo surgery (5 [46%] nonsurvivors vs. 11 [69%] survivors, p = 0.26) (Table 4). SOFA score calculated within the first 24 hours after patients sought care was higher for nonsurvivors than for survivors and for only those requiring ICU admission (p<0.003) (Figure 4).

**Table 3:** Initial administration of antibacterial drugs to persons who inject drugs and had *Bacillus anthracis* Infection, Scotland, UK, 2009–2010*

Patient no.†	CLI	CIP	BPC	FLUX	MTZ	CRO	GEN	VAN	MEM	TZP	AMC	AMX
Survivor, n = 16‡	14 (88)	12 (75)	11 (69)	11 (69)	8 (50)	1 (6)	0	2 (13)	1 (6)	0	1 (6)	0
1	+	+	–	–	+	–	–	+	–	–	–	–
2	+	–	–	+	+	–	–	–	–	–	–	–
3	–	–	+	+	–	–	–	–	–	–	+	–
4	+	+	+	+	+	–	–	–	–	–	–	–
5	+	+	+	–	+	–	–	–	–	–	–	–
6	+	+	+	–	–	–	–	–	–	–	–	–
7	+	+	+	+	+	–	–	–	–	–	–	–
8	+	+	+	+	–	–	–	–	–	–	–	–
9	+	+	+	+	+	–	–	–	–	–	–	–
10	+	+	+	+	–	–	–	–	–	–	–	–
11	–	–	–	+	–	–	–	–	–	–	–	–
12	+	+	+	+	+	–	–	–	–	–	–	–
13	+	–	–	–	–	–	–	–	+	–	–	–
14	+	+	+	+	–	–	–	–	–	–	–	–
15	+	+	+	+	+	+	–	–	–	–	–	–
16	+	+	–	–	–	–	–	+	–	–	–	–
Nonsurvivor, n = 11‡	7 (64)	6 (55)	5 (45)	5 (45)	6 (55)	4 (36)	3 (27)	1 (9)	1 (9)	1 (9)	0	1 (9)
1	–	–	–	–	+	+	–	–	–	–	–	–
2	–	–	–	–	–	+	–	–	–	–	–	–
3	+	+	+	+	+	–	+	–	–	–	–	–
4	+	+	+	+	+	–	–	–	–	–	–	–
5	–	–	–	+	–	–	–	–	–	–	–	–
6	+	+	+	+	+	–	–	–	–	–	–	–

7	+	+	+	–	–	–	–	–	–	–	–	–
8	+	–	+	+	–	–	+	–	–	–	–	–
9	+	+	–	–	+	+	+	+	+	+	–	–
10	+	+	–	–	+	+	–	–	–	–	–	–
11	–	–	–	–	–	–	–	–	–	–	–	+
Total, n = 27	21 (78)	18 (67)	16 (59)	16 (59)	14 (52)	5 (19)	3 (11)	3 (11)	2 (7)	1 (4)	1 (4)	1 (4)

*CLI, clindamycin; CIP, ciprofloxacin; BPC, benzylpenicillin (penicillin G); FLUX, flucloxacillin; MTZ, metronidazole; CRO, ceftriaxone; GEN, gentamicin; VAN, vancomycin; MEM, meropenem; TZP, piperacillin/tazobactam; AMC, amoxicillin/clavulanic acid; AMX, amoxicillin; +, antibacterial drug administered; –, antibacterial drug not administered. †Patients for whom data were available are numbered in the order in which they sought care during the outbreak. ‡No. (%) patients receiving an antibacterial drug.

**Figure 4:** Treatments at time patients sought care, SOFA scores, and treatments anytime during hospitalization of persons who inject drugs and were part of an outbreak of *Bacillus anthracis* infection, Scotland, UK, 2009–2010. Included are the 27 patients for whom data were available on treatment with vasopressors (A), oxygen therapy (B), mechanical ventilation (C), or steroids (D); the mean (+ SEM) SOFA score calculated within the first 24 h of hospitalization in all patients (E) and in only those who required ICU admission (F); and the proportion of patients at time they sought care who required ICU admission (G) or were treated with vasopressors (H), mechanical ventilation (I), or anthrax immune globulin (J) at any time during hospitalization. For panels A–D and G–J, n = the number of survivors or nonsurvivors receiving treatment/total number for whom data were available. For panels E and F, n = the number of survivors or nonsurvivors for whom SOFA scores were calculated. SOFA, sequential organ failure assessment; ICU, intensive care unit.

**Table 4:** Types of initial surgery and tissue findings on gross examination for persons who inject drugs and had *Bacillus anthracis* Infection, Scotland, UK, 2009–2010*

Patient no.†	Surgery						Gross tissue findings at surgery				
	Debride	Fasciotomy	Thigh ex	Laparotomy	Incision drainage		Excessive edema	Necrotic tissue	Excessive bleeding	Fasciitis	Liquefied necrosis
Survivor‡	5/11 (45)	4/11 (36)	1/11 (9)	0/11 (0)	1/11 (9)		5/9 (56)	7/11 (64)	1/11 (9)	1/10 (10)	1/10 (10)
1	–	–	+	–	–		+	–	+	–	–
2	–	–	–	–	+		–	+	–	–	+
3	+	–	–	–	–		+	+	–	–	–
4	+	–	–	–	–		–	+	–	–	–
5	–	+	–	–	–		+	–	–	–	–
7	+	–	–	–	–		+	+	–	–	–
10	–	+	–	–	–		–	–	–	–	–
12	+	–	–	–	–		NA	+	–	+	–
13	+	–	–	–	–		+	+	–	–	–
14	–	+	–	–	–		–	–	–	–	–
15	–	+	–	–	–		NA	+	–	NA	NA
Nonsurvivor‡	4/5 (80)	0/5	0/5	1/5 (20)	0/5 (0)		5/5 (100)	3/5 (60)	4/5 (80)§	2/4 (50)	1/4 (25)
3	+	–	–	–	–		+	+	+	+	–
4	+	–	–	–	–		+	+	+	+	–
6	+	–	–	–	–		+	+	+	NA	NA
8	+	–	–	–	–		+	–	–	–	–
9	–	–	–	+	–		+	–	+	–	+
Total	9/16 (56)	4/16 (25)	1/16 (6)	1/16 (6)	1/16 (6)		10/14 (71)	10/16 (63)	5/16 (31)	3/14 (21)	2/14 (14)

*Debride, debridement; thigh ex., thigh exploration; +, type of surgery or tissue finding performed or noted; –, type of surgery or tissue finding not performed or noted; NA, observation not available. †Patients are numbered in the order in which they sought care. ‡No. (%) patients with the type of surgery or finding/total no. patients for whom data were reported. §For proportion of positive responses by survivors vs. nonsurvivors for whom data were reported, p<0.05.

### Confirmation of *B. anthracis* Infection

The basis for the microbiological diagnosis of *B. anthracis* for the 47 confirmed anthrax cases has been published (*3*). For the 27 cases reported here, the median (IQR) time (days) to confirmation was 3 (1.5–9.0) and did not differ between nonsurvivors and survivors (3.0 [2.0–7.0] vs. 3.0 [1.0–9.0], respectively, p = 0.83) (Figure 1). Results of tests confirming *B. anthracis* infection did not differ significantly between survivors and nonsurvivors (p>0.05 for all) (Table 2). We had insufficient data to determine how often bacteria other than *B. anthracis* were found in blood or tissue samples.

### Hospital Course

Proportionately more nonsurvivors than survivors received ICU care (11 [100%] vs. 7 [44%], p = 0.003) (Figure 4). The median (IQR) time (days) survivors remained in the ICU and hospital were 2.0 (1.0–13.3) and 21.5 (4.5–35.5), respectively. For nonsurvivors, median time from hospital admission to death was 2.1 (0.63–4) days. During hospitalization, proportionately more nonsurvivors received vasopressors (p = 0.0001) (Figure 4). More nonsurvivors also required mechanical ventilation (p = 0.005). Among all patients, 11 (79%) of the 14 who received vasopressors at any time died. Twelve (44%) of the 27 patients received anthrax immune globulin. We found no significant difference in the proportions of survivors and nonsurvivors who received anthrax immune globulin (p = 0.93, Figure 4) or in the median (IQR) time (days) to treatment (3 [1–3] vs. 1 [1–1]), respectively; p = 0.13).

We found no significant difference in the proportions of survivors and nonsurvivors who required surgery during hospitalization (11 [69%] vs. 5 [46%]; p = 0.26) or who required >1 surgery (7 [64%] vs. 4 (80%); p = 0.52) or in the median (IQR) time (days) to initial surgery (0 [0–1] vs. 0 [0–0.125]; p = 0.53) (Figure 1). Even when we examined data from the first week of hospitalization only, survivors and nonsurvivors did not differ significantly in the proportion having surgery (7 [44%] survivors vs. 4 [36%] nonsurvivors; p = 0.56). However, nonsurvivors more often bled excessively during surgery (4 [80%] nonsurvivors vs. 1 [8%] survivor; p = 0.01) (Table 4). One survivor had an arm amputated above the elbow, and 4 received skin grafts.

Receipt of packed erythrocytes, fresh frozen plasma, cryoglobulin, and platelets; renal replacement therapy; and pleural or peritoneal drainage did not differ significantly between survivors and nonsurvivors (Table 2). For 9 (36%) of 25 patients for whom information was available, cardiac function was assessed: echocardiography for 4 patients, troponin measures for 3, and lithium dilution cardiac output and pulse contour cardiac output for 1 each. Of these, 3 nonsurvivors were noted to have evidence of myocardial dysfunction on the basis of echocardiography, lithium dilution cardiac output, or pulse contour cardiac output; 1 survivor had an elevated troponin level. Causes of death for the 7 patients for whom autopsies were reported were as follows; multiple system organ failure caused by *B. anthracis* sepsis, 2 patients; necrotizing fasciitis related to *B. anthracis*, 1; sepsis and hemorrhagic meningitis with *B. anthracis* infection, 2; subarachnoid hemorrhage, 1; and myocardial infarction, 1.

## Discussion

Our review of 27 confirmed cases of *B. anthracis* infection in PWID compares clinical findings in survivors and nonsurvivors of this newly described form of infection. Although duration of symptoms and time to seeking hospital care did not differ between survivors and nonsurvivors, the severity of illness did. Most survivors reported localized symptoms related to the injection site, and none required vasopressor therapy or mechanical ventilation. In contrast, most nonsurvivors had generalized symptoms and evidence of sepsis, which required both vasopressor support and mechanical ventilation. Nonsurvivors also had lower systolic blood pressures and Glasgow coma scores; higher respiratory rates; worsened base deficits; higher levels of hemoglobin (consistent with hemoconcentration) and C-reactive protein; higher international normalized ratio; and lower sodium and albumin levels and platelet counts. During hospitalization, all nonsurvivors required vasopressor and ICU support, whereas only 3 and 7 survivors, respectively, required these. SOFA scores were substantially higher in nonsurvivors than survivors. Thus, assessing the need for aggressive cardiopulmonary support or determining a score like SOFA for patients with injectional anthrax can help identify those for whom prognosis is particularly poor and more aggressive therapy is needed.

Possibly consistent with prior analysis showing an association between excessive alcohol use and risk for *B. anthracis* infection in PWID, we found a higher incidence of excessive alcohol use among nonsurvivors than survivors (*6*). Increased bilirubin and decreased albumin levels in nonsurvivors might in part have reflected preexisting alcoholic liver disease. Although age did not differ significantly between survivors and nonsurvivors, the latter tended to be older, a finding consistent with analysis of inhalational *B. anthracis* infection (*14*).

Differences in outcome between survivors and nonsurvivors did not appear related to variation in treatment. All patients received antibacterial drugs from the time they sought care, and the types and numbers of antibacterial drugs administered did not differ. Also, the proportion of patients who had >1 surgeries and the time from admission to initial surgery did not differ. Finally, similar proportions of survivors and nonsurvivors received anthrax immune globulin, and the median time to treatment for these groups did not differ.

The most common skin and limb findings were localized edema, pain, and erythema. Although these findings are consistent with soft tissue infection, their presence did not differ between survivors and nonsurvivors (*15*). Thus, skin and limb findings and even the need for surgery did not appear to predict worsened prognoses. Notably absent in most patients was the eschar formation classically associated with cutaneous *B. anthracis* infection (*16*,*17*). This absence combined, with the frequent need for surgery and the overall high death rate despite receipt of antibacterial drugs, supports the observation that the pathogeneses of injectional and cutaneous anthrax differ (*3*,*4*–*6*).

In general, the severity of soft tissue infection and its requirement for surgery varies from mild to severe on the basis of the depth of tissue involvement and tissue necrosis (*15*). The *B. anthracis* soft tissue infections reviewed here reflect this range of disease. Two survivors had symptoms consistent with cellulitis, were treated only with antibacterial drugs, and were discharged within 1–2 days. However, 10 survivors and 5 nonsurvivors required debridement, fasciotomy, or laparotomy on >1 occasion; 10 had evidence of tissue necrosis. Even among the 5 survivors who did not have surgery, 3 required hospitalization for >7 days, which suggests severe infection. Without additional data about co-existing conditions, we cannot determine whether outcomes from soft tissue infection in PWID differ between *B. anthracis* and other bacteria.

*B. anthracis* has a cell wall that elicits a robust host inflammatory response and the endothelial dysfunction, shock, and organ injury with which this response is associated (*1*,*18*–*21*). However, *B. anthracis* also releases lethal and edema toxins that can produce this same dysfunction but through very different mechanisms than the cell wall (*22*,*23*). Whether, as a result of these diverse nontoxin and toxin components, the manifestations of soft tissue infection with *B. anthracis* differ from those of other bacteria is unclear. Several differences between nonsurvivors and survivors, such as reduced systolic blood pressure and sodium and worsened acidosis, are associated with worsened outcome with other types of soft tissue infection (*24*–*26*). However, of patients who required vasopressor treatment, almost 80% died. This death rate is high, even for patients identified with septic shock on the basis of need for vasopressors. However, this finding is consistent with the 2001 US outbreak of inhalational *B. anthracis* in which all patients in whom shock developed died (*27*). Also in the current review, nonsurvivors bled more during surgery, possibly because of an increase in international normalized ratio and a decrease in platelets. Although excessive bleeding is not typically associated with soft tissue infection, it is associated with inhalational and gastrointestinal *B. anthracis* infection (*28*,*29*).

*B. anthracis* lethal and edema toxins inhibit components in the innate and adaptive immune responses (*22*,*23*). This inhibition might contribute to infection, as well as suppress signs typically associated with an activated host inflammatory response (*3*). However, although temperature was lower in nonsurvivors than in survivors, circulating leukocyte counts, percentage of neutrophils, and C-reactive protein were higher and in ranges approaching or consistent with invasive soft tissue infection caused by other bacteria (*30*,*31*). Whether toxin production interferes with host defense and influences the features and course of injectional *B. anthracis* infection requires further study.

Several lines of evidence suggest that *B. anthracis* lethal toxin produces direct cardiac dysfunction (*22*). Whether such dysfunction contributes to clinical *B. anthracis* infection is unclear because there are few measures of cardiac function in patients. Although 4 of the 9 patients in the current review were described as having evidence of cardiac dysfunction, these data were limited. More comprehensive investigation of cardiac function is necessary during future outbreaks of *B. anthracis*.

This study has limitations. First, data were collected >2 years after patients sought care, were not obtainable for some questions on patients we included in the analysis, and were unavailable for 20 of the 47 confirmed cases. However, these 27 patients included 11 of the 14 nonsurvivors from the outbreak and probably were fairly representative of nonsurvivors. Second, data were limited regarding co-morbidities, particularly HIV infection and viral hepatitis status, which might have influenced outcome. Information about other co-morbidities (e.g., diabetes, heart disease, and chronic lung disease) might have been informative as well. Third, although 1 survivor and 1 nonsurvivor described headache, using the Glasgow coma scale to assess neurologic status might not have captured other patients with this symptom, a possible manifestation of underlying meningeal infection. Fourth, comparisons of therapies administered to survivors and nonsurvivors later than when they initially sought care might have been confounded by patients’ length of hospitalization; however, data were insufficient to analyze the influence of this variable. Finally, autopsy findings were available only for 7 patients.

The 2009–2010 outbreak of *B. anthracis* infection among PWID in Scotland was considered over at the end of 2010 (*3*). However, during June 2012–December 2013, thirteen new cases were reported in the United Kingdom and Europe (*4*,*32*). The death rate for these patients has been close to 50%. Findings from the patients in the current review, combined with findings from newer cases, emphasize the need to better understand the pathogenesis and management of this recently identified form of *B. anthracis* infection.

## References

[1] Sweeney DA, Hicks CW, Cui X, Li Y, Eichacker PQ. Anthrax infection. Am J Respir Crit Care Med. 2011;184:1333–41 and. 10.1164/rccm.201102-0209CI21852539PMC3361358

[2] Booth MG, Hood J, Brooks TJ, Hart A. Anthrax infection in drug users. Lancet. 2010;375:1345–6 and. 10.1016/S0140-6736(10)60573-920399978

[3] Health Protection Scotland. An outbreak of anthrax among drug users in Scotland, December 2009 to December 2010. A report on behalf of the National Anthrax Outbreak Control Team [cited 2013 May 30]. http://www.documents.hps.scot.nhs.uk/giz/anthrax-outbreak/anthrax-outbreak-report-2011-12.pdf

[4] Grunow R, Klee SR, Beyer W, George M, Grunow D, Barduhn A, Anthrax among heroin users in Europe possibly caused by same Bacillus anthracis strain since 2000. Euro Surveill. 2013;18:20437.23557972

[5] Ringertz SH, Hoiby EA, Jensenius M, Maehlen J, Caugant DA, Myklebust A, Injectional anthrax in a heroin skin-popper. Lancet. 2000;356:1574–5 . 10.1016/S0140-6736(00)03133-011075776

[6] Palmateer NE, Ramsay CN, Browning L, Goldberg DJ, Hutchinson SJ. Anthrax infection among heroin users in Scotland during 2009–2010: a case–control study by linkage to a national drug treatment database. Clin Infect Dis. 2012;55:706–10 . 10.1093/cid/cis51122618565

[7] Price EP, Seymour ML, Sarovich DS, Latham J, Wolken SR, Mason J, Molecular epidemiologic investigation of an anthrax outbreak among heroin users, Europe. Emerg Infect Dis. 2012;18:1307–13. 10.3201/eid1808.11134322840345PMC3414016

[8] Beaumont G. Anthrax in a Scottish intravenous drug user. J Forensic Leg Med. 2010;17:443–5 and. 10.1016/j.jflm.2010.09.00821056883

[9] Jallali N, Hettiaratchy S, Gordon AC, Jain A. The surgical management of injectional anthrax. J Plast Reconstr Aesthet Surg. 2011;64:276–7. 10.1016/j.bjps.2010.06.00320615773

[10] Johns N, Cooper D, Terrace J. An unusual case of peritonitis in an intravenous drug user. Gastroenterology. 2011;141:435–6, 780–1. 10.1053/j.gastro.2011.02.07621712037

[11] Knox D, Murray G, Millar M, Hamilton D, Connor M, Ferdinand RD, Subcutaneous anthrax in three intravenous drug users: a new clinical diagnosis. J Bone Joint Surg Br. 2011;93:414–7. 10.1302/0301-620X.93B3.2597621357967

[12] Parcell BJ, Wilmshurst AD, France AJ, Motta L, Brooks T, Olver WJ. Injection anthrax causing compartment syndrome and necrotising fasciitis. J Clin Pathol. 2011;64:95–6. 10.1136/jcp.2010.08258621097792

[13] Vincent JL, Moreno R, Takala J, Willatts S, De Mendonca A, Bruining H, The SOFA (sepsis-related organ failure assessment) score to describe organ dysfunction/failure. On behalf of the Working Group on Sepsis-Related Problems of the European Society of Intensive Care Medicine. Intensive Care Med. 1996;22:707–10. 10.1007/BF017097518844239

[14] Holty JE, Bravata DM, Liu H, Olshen RA, McDonald KM, Owens DK. Systematic review: a century of inhalational anthrax cases from 1900 to 2005. Ann Intern Med. 2006;144:270–80. 10.7326/0003-4819-144-4-200602210-0000916490913

[15] Ebright JR, Pieper B. Skin and soft tissue infections in injection drug users. Infect Dis Clin North Am. 2002;16:697–712. 10.1016/S0891-5520(02)00017-X12371123

[16] Doganay L, Welsby PD. Anthrax: a disease in waiting? Postgrad Med J. 2006;82:754–6 . 10.1136/pgmj.2005.04448717099096PMC2660504

[17] Doganay M, Metan G, Alp E. A review of cutaneous anthrax and its outcome. J Infect Public Health. 2010;3:98–105. 10.1016/j.jiph.2010.07.00420869669

[18] Cui X, Su J, Li Y, Shiloach J, Solomon S, Kaufman JB, *Bacillus anthracis* cell wall produces injurious inflammation but paradoxically decreases the lethality of anthrax lethal toxin in a rat model. Intensive Care Med. 2010;36:148–56. 10.1007/s00134-009-1643-919756496PMC3401931

[19] Langer M, Malykhin A, Maeda K, Chakrabarty K, Williamson KS, Feasley CL, *Bacillus anthracis* peptidoglycan stimulates an inflammatory response in monocytes through the p38 mitogen-activated protein kinase pathway. PLoS ONE. 2008;3:e3706. 10.1371/journal.pone.000370619002259PMC2577892

[20] Popov SG, Villasmil R, Bernardi J, Grene E, Cardwell J, Popova T, Effect of *Bacillus anthracis* lethal toxin on human peripheral blood mononuclear cells. FEBS Lett. 2002;527:211–5. 10.1016/S0014-5793(02)03228-312220662

[21] Qiu P, Li Y, Shiloach J, Cui X, Sun J, Trinh L, *Bacillus anthracis* cell wall peptidoglycan but not lethal or edema toxins produces changes consistent with disseminated intravascular coagulation in a rat model. J Infect Dis. 2013;208:978–89. 10.1093/infdis/jit24723737601PMC3749004

[22] Hicks CW, Cui X, Sweeney DA, Li Y, Barochia A, Eichacker PQ. The potential contributions of lethal and edema toxins to the pathogenesis of anthrax associated shock. Toxins. 2011;3:1185–202.10.3390/toxins3091185PMC320287722069762

[23] Moayeri M, Leppla SH. Cellular and systemic effects of anthrax lethal toxin and edema toxin. Mol Aspects Med. 2009;30:439–55. 10.1016/j.mam.2009.07.00319638283PMC2784088

[24] Dworkin MS, Westercamp MD, Park L, McIntyre A. The epidemiology of necrotizing fasciitis including factors associated with death and amputation. Epidemiol Infect. 2009;137:1609–14. 10.1017/S095026880900253219351432

[25] Frazee BW, Fee C, Lynn J, Wang R, Bostrom A, Hargis C, Community-acquired necrotizing soft tissue infections: a review of 122 cases presenting to a single emergency department over 12 years. J Emerg Med. 2008;34:139–46. 10.1016/j.jemermed.2007.03.04117976799

[26] Yaghoubian A, de Virgilio C, Dauphine C, Lewis RJ, Lin M. Use of admission serum lactate and sodium levels to predict mortality in necrotizing soft-tissue infections. Arch Surg. 2007;142:840–6, discussion 4–6. 10.1001/archsurg.142.9.84017875838

[27] Sherer K, Li Y, Cui X, Eichacker PQ. Lethal and edema toxins in the pathogenesis of *Bacillus anthracis* septic shock: implications for therapy. Am J Respir Crit Care Med. 2007;175:211–21 . 10.1164/rccm.200608-1239CP17095744PMC2176088

[28] Abramova FA, Grinberg LM, Yampolskaya OV, Walker DH. Pathology of inhalational anthrax in 42 cases from the Sverdlovsk outbreak of 1979. Proc Natl Acad Sci U S A. 1993;90:2291–4. 10.1073/pnas.90.6.22918460135PMC46072

[29] Grinberg LM, Abramova FA, Yampolskaya OV, Walker DH, Smith JH. Quantitative pathology of inhalational anthrax I: quantitative microscopic findings. Mod Pathol. 2001;14:482–95. 10.1038/modpathol.388033711353060

[30] Chan T, Yaghoubian A, Rosing D, Kaji A, de Virgilio C. Low sensitivity of physical examination findings in necrotizing soft tissue infection is improved with laboratory values: a prospective study. Am J Surg. 2008;196:926–30, discussion 30. 10.1016/j.amjsurg.2008.07.02519095111

[31] Su YC, Chen HW, Hong YC, Chen CT, Hsiao CT, Chen IC. Laboratory risk indicator for necrotizing fasciitis score and the outcomes. ANZ J Surg. 2008;78:968–72. 10.1111/j.1445-2197.2008.04713.x18959694

[32] Holzmann T, Frangoulidis D, Simon M, Noll P, Schmoldt S, Hanczaruk M, Fatal anthrax infection in a heroin user from southern Germany, June 2012. Euro Surveill. 2012;17:20204 .22790532

# Review article

## Medscape CME Quiz

## Earning CME Credit

To obtain credit, you should first read the journal article. After reading the article, you should be able to answer the following, related, multiple-choice questions. To complete the questions (with a minimum 75% passing score) and earn continuing medical education (CME) credit, please go to www.medscape.org/journal/eid. Credit cannot be obtained for tests completed on paper, although you may use the worksheet below to keep a record of your answers. You must be a registered user on Medscape.org. If you are not registered on Medscape.org, please click on the “Register” link on the right hand side of the website to register. Only one answer is correct for each question. Once you successfully answer all post-test questions you will be able to view and/or print your certificate. For questions regarding the content of this activity, contact the accredited provider, CME@medscape.net. For technical assistance, contact CME@webmd.net. American Medical Association’s Physician’s Recognition Award (AMA PRA) credits are accepted in the US as evidence of participation in CME activities. For further information on this award, please refer to http://www.ama-assn.org/ama/pub/about-ama/awards/ama-physicians-recognition-award.page. The AMA has determined that physicians not licensed in the US who participate in this CME activity are eligible for *AMA PRA Category 1 Credits™*. Through agreements that the AMA has made with agencies in some countries, AMA PRA credit may be acceptable as evidence of participation in CME activities. If you are not licensed in the US, please complete the questions online, print the certificate and present it to your national medical association for review.

## Article Title:  Confirmed *Bacillus anthracis* Infection among Persons Who Inject Drugs, Scotland, 2009–2010

## CME Questions

**1. You are seeing a 33-year-old man in the emergency department. He complains of severe pain and redness on his left upper extremity after injecting heroin into an antecubital vein yesterday. A brief examination of the area reveals 8 cm of erythema, induration, and calor, with a central necrotic area. You consider whether this patient has injection anthrax; you also consider his prognosis. Which one of the following patient factors was most associated with a higher risk for mortality related to injection anthrax in the current study?**A. Younger ageB. Male sexC. Excessive alcohol useD. Cigarette smoking**2. Which one of the following variables at patient presentation was most associated with a higher risk for mortality in the current study of patients with injection anthrax?**A. Temperature less than 36˚CB. Heart rate greater than 115 beats per minuteC. Respiratory rate less than 12 breaths per minuteD. Skin lesions on the upper extremity vs lower extremity**3. Which one of the following statements regarding treatment and mortality outcomes related to injection anthrax in the current study is most accurate?**A. Rates of initial diagnosis with *Bacillus anthracis* infection were similar among nonsurvivors and survivorsB. Compared with survivors, nonsurvivors were less likely to receive antibiotics at hospital admissionC. Compared with survivors, nonsurvivors were less likely to receive ciprofloxacin D. Nonsurvivors and survivors were equally likely to require surgery**4. Which one of the following hospital events was more common among nonsurvivors vs survivors in the current study?**A. Application of anthrax immune globulinB. Excessive bleeding during surgeryC. Longer duration until initial surgeryD. Initiation of renal replacement therapy

## Activity Evaluation

**1:** The activity supported the learning objectives.

Strongly Disagree				Strongly Agree
1	2	3	4	5
**2. The material was organized clearly for learning to occur.**				
Strongly Disagree				Strongly Agree
1	2	3	4	5
**3. The content learned from this activity will impact my practice.**				
Strongly Disagree				Strongly Agree
1	2	3	4	5
**4. The activity was presented objectively and free of commercial bias.**				
Strongly Disagree				Strongly Agree
1	2	3	4	5
